# Enhancing Healthcare Services Quality in a Resource–Limited Country: Perspectives From Cambodia

**DOI:** 10.1002/hsr2.72373

**Published:** 2026-04-15

**Authors:** Virak Sorn

**Affiliations:** ^1^ Faculty of Health Sciences and Biotechnology University of Puthisastra Cambodia

**Keywords:** Cambodia, public health reform, quality improvement, social health protection, universal health coverage (UHC)

## Abstract

**Background and Aim:**

Achieving Universal Health Coverage (UHC) is a global priority focused on ensuring access to high‐quality healthcare services. Cambodia is committed to improving healthcare quality and expanding social health protection through several strategic initiatives. However, enhancing the quality of healthcare in resource‐limited settings remains a significant challenge due to factors such as constrained financial resources, workforce shortages, and inadequate infrastructure. This perspective aims to examine the key challenges and critical areas for improvement in Cambodia's healthcare system. It also explores the enablers and barriers to initiating, implementing, and sustaining healthcare quality improvements in the country.

**Discussion:**

Cambodia's healthcare system has made progress over the past three decades. However, significant challenges persist, particularly in the quality of healthcare services and public trust in the system. Improving healthcare quality will require strategic investments in infrastructure, governance, and workforce development. Additionally, innovative approaches—such as public‐private partnerships and digital health technologies—are essential in resource‐constrained settings. Policy reforms, enhanced community‐based health initiatives, and capacity‐building efforts are also necessary to accelerate progress toward UHC in Cambodia.

**Conclusions:**

This perspective underscores the need for a comprehensive, multisectoral approach to improve healthcare quality in Cambodia. Addressing issues related to governance, financing, infrastructure, and human resources is essential. Strategic measures such as public‐private partnerships, capacity‐building initiatives, health equity funds, and the integration of digital health technologies hold promise for advancing UHC in the Cambodian context.

## 1 Background

Sustainable Development Goal 3 (SDG 3) includes the target of achieving Universal Health Coverage (UHC), which ensures financial protection and access to essential, high‐quality health services and medications [1]. As emphasized by Kruk et al. in *The Lancet Global Health Commission on High‐Quality Health Systems in the SDG Era*, improving healthcare quality is a fundamental component of UHC in all countries [2]. Cambodia's healthcare system has made notable progress over recent decades, particularly in expanding access to essential health services [3]. However, significant challenges remain, especially in resource‐limited settings where financial constraints, workforce shortages, and inadequate infrastructure continue to impede the delivery of high‐quality care [4, 5, 6].

Currently, approximately 54% of Cambodians lack any form of health coverage, and only 30–40% are covered by social health protection mechanisms [3, 6, 7]. Public health services remain underfunded, with rural populations facing major barriers to accessing essential care [8]. Meanwhile, the private sector has become increasingly prominent in healthcare provision; however, concerns about service quality, affordability, and insufficient regulatory oversight persist [8, 9, 10].

Enhancing healthcare quality is vital to achieving UHC and ensuring equitable access to care for all individuals [10]. Although quality of care is widely recognized as a key element in health system strengthening, the evolving nature of healthcare delivery demands a deliberate focus on service quality [9, 10, 11, 12]. Strong leadership and effective governance are essential to building a robust quality management infrastructure [12]. Additionally, external incentives and accountability mechanisms can drive improvements in both quality and performance [13]. Improving the quality of healthcare services is not only critical for achieving better health outcomes but also for strengthening public trust in the health system, reducing health disparities, and ensuring efficient use of resources [2, 8]. Without targeted interventions to address persistent healthcare services quality gaps, Cambodia's progress toward UHC will remain slow and uneven. This paper, therefore, examines the key challenges impacting healthcare services quality in Cambodia and explores strategic approaches for improvement. It highlights core issues such as limited financing, workforce capacity constraints, governance weaknesses, and infrastructure deficits. Furthermore, it discusses innovative solutions—including public‐private partnerships, digital health technologies, and community‐based initiatives—that can help enhance service quality and build a more resilient and equitable healthcare system in Cambodia.

## 2 Current State of Health Services Quality in Cambodia

Cambodia has made notable progress in key health indicators over the past three decades, yet significant challenges remain in achieving high‐quality healthcare services. In 2021, Cambodia's UHC service coverage index stood at 58, compared to 68 for Vietnam, 82 for Thailand, 89 for Singapore and South Korea, 83 for Japan, and 81 for China. The average UHC score for the East Asia and Pacific region was 75 [14]. While life expectancy in Cambodia is 70.74 years [15], disparities in health outcomes persist across caste, region, gender, class, and geography. The country continues to report an infant mortality rate of 20.17 per 1000 live births [16] and a maternal mortality rate of 218 per 100,000 live births [17], respectively.

Cambodia's healthcare system operates as a mixed public‐private model, with both sectors playing vital roles in service delivery. The public healthcare system, overseen by the Ministry of Health (MoH), offers essential services through a network of national hospitals, provincial and district referral hospitals, and health centers [6]. These public services aim to be affordable and accessible, particularly for low‐income populations [18, 19]. However, public health facilities frequently face challenges such as underfunding, insufficient medical supplies, and workforce shortages—factors that compromise service quality and efficiency [4, 8, 18, 19, 20]. In contrast, the private healthcare sector has expanded rapidly, offering services ranging from basic care to specialized treatments. Private providers are often preferred due to shorter wait times, a perception of higher service quality, and greater availability of medical specialists. However, the sector remains largely unregulated, raising concerns about medical ethics, affordability, and disparities in access between urban and rural areas [8]. A substantial portion of the population continues to rely on out‐of‐pocket payments (55%), which often result in financial hardship, particularly for the poor [18, 19].

With a population of 17 million, Cambodia reported an annual per capita health expenditure of approximately $122 in 2021. The country also faces persistent challenges related to an undertrained and unevenly distributed healthcare workforce. In 2012, Cambodia fell below the WHO's critical shortage threshold, with only 1.4 health workers per 1000 people. Moreover, one‐half of the country's doctors and three‐quarters of medical specialists are concentrated in the Phnom Penh capital, while approximately 75% of the population resides in rural areas [4]. As a result, infant mortality in rural areas is three times higher than in urban centers [8]. Despite the fact that over 80% of the population lives within 5 km of a healthcare facility, access to services remains limited due to cost barriers and concerns about care quality. Out‐of‐pocket healthcare spending continues to be high, further impeding access for low‐income populations [18, 19, 20].

To address the issues outlined above, the Cambodian government has implemented several initiatives aimed at enhancing healthcare quality and achieving Universal Health Coverage (UHC). These include the Health Equity Fund (HEF), which provides financial support to low‐income populations by covering healthcare costs at public facilities [8, 18, 19, 20, 21, 22]; the National Health Strategic Plan (NHSP), which focuses on improving service quality, expanding health workforce training, and strengthening health infrastructure [6, 8]; and Social Health Protection Programs (SHPP), which aim to expand health insurance coverage, including through the National Social Security Fund (NSSF) for formal sector workers [3, 6, 7, 8, 18, 19, 20, 21, 22]. Additional efforts include the regulation of private healthcare, with steps taken to improve licensing, accreditation, and monitoring of private health providers to ensure quality and affordability [2], and the promotion of digital health initiatives, such as the adoption of telemedicine and electronic health records, to improve service efficiency, particularly in rural areas [10, 23, 24]. While these policies have contributed to improvements in the health system, persistent challenges—such as inadequate funding, workforce shortages, and regulatory gaps—continue to hinder the overall quality of healthcare services. Strengthening governance, expanding social health protection, and integrating innovative healthcare solutions remain crucial for sustainable progress.

## 3 Key Challenges in Enhancing Healthcare Services Quality

### 3.1 Limited Financial Resources

A major barrier to improving healthcare quality in Cambodia is insufficient financial investment in the health sector. As of 2021, public health expenditure remained low, with over 55% of healthcare financing coming from out‐of‐pocket payments by patients, which can push families into poverty and prevent timely care for those who cannot afford it [25]. This underfunding directly impacts the availability of essential medicines, medical equipment, and facility maintenance. On the other hand, this will have detrimental impacts on the personnel, decreasing morale among healthcare workers due to lower incomes, poor working conditions, and a lack of motivation among healthcare professionals [18, 19, 20, 26]. Consequently, public health institutions often struggle to provide consistent, high‐quality services—particularly in rural areas, where financial constraints are more pronounced [2, 27].

### 3.2 Workforce Shortages

Cambodia faces a critical shortage of trained healthcare professionals, including doctors, nurses, and specialists. In 2012, Cambodia had only 1.4 health workers per 1000 population—well below the World Health Organization's recommended threshold of 4.5 health workers per 1000 population for adequate service coverage. Although the ratio improved to 2.0 per 1000 by 2023, it still remains below the ASEAN average. The government aims to increase this figure to 2.4 per 1000 by 2030 [27]. Additionally, retention remains a major issue, especially in rural and underserved areas, where healthcare professionals often migrate to urban centers or enter into the private sector in search of better compensation and working conditions [4, 8]. This imbalance leads to overburdened staff, diminished quality of care, and prolonged wait times for patients [8, 28].

### 3.3 Infrastructural Deficits

Over the past thirty years, Cambodia has made substantial investments and advancements in its healthcare system, particularly through public health reforms, donor‐funded programs, and the expansion of infrastructure and human resources after the civil war, the Cambodian–Vietnamese war, and prolonged insurgency by the Khmer Rouge, which ended in 1975 and 1999. Notably, the government increased its national health budget and developed the Health Sector Strategic Plans (HSP I–IV), focusing on expanding primary health care, maternal and child health services, and controlling communicable diseases [29]. International partnerships with organizations such as the World Health Organization (WHO), the Asian Development Bank (ADB), and USAID have supported the construction and rehabilitation of health centers and referral hospitals, the training of healthcare workers, and the implementation of health information systems [18, 19, 20, 21, 22, 29]. Cambodia also introduced the Health Equity Fund (HEF) to provide financial protection for the poor and scaled up vaccination programs and disease surveillance systems [18, 19, 20, 21, 22]. Despite significant investments over the past three decades, many healthcare facilities in Cambodia—particularly in rural regions—continue to lack essential infrastructure and medical equipment advancement [6, 30]. Numerous public health centers operate with outdated tools, insufficient hospital beds, and limited access to medications and diagnostic technologies [6]. Poor transportation infrastructure and road conditions further hinder timely access to care for rural populations. Inadequate sanitation and electricity in some facilities also compromise patient safety and service delivery efficiency [31].

### 3.4 Governance and Policy Gaps

Cambodia faces significant challenges in its health sector, including governance and policy gaps that hinder the efficiency, equity, and quality of healthcare services. Weak governance and regulatory shortcomings remain major obstacles to healthcare quality, as oversight of both public and private healthcare providers is often inconsistent, resulting in variations in service standards across the country [32]. The lack of a comprehensive national public health law has left regulatory frameworks weak, particularly in areas like health professional licensing and facility accreditation. While corruption and lack of accountability in health financing, procurement, and staffing further compromise service delivery [33, 34]. Additionally, the widespread dual practice of public‐sector health professionals working in private clinics diverts time and resources away from the public health system [32, 34]. Limited citizen participation, weak health information systems, and insufficient data transparency hinder evidence‐based policy decisions and long‐term planning. Furthermore, the lack of robust accreditation systems for private clinics and hospitals has led to unregulated medical practices, raising concerns about patient safety and further eroding public trust in healthcare services [35].

### 3.5 Health Equity Concerns

Significant disparities in healthcare access persist between urban and rural populations. While cities such as Phnom Penh benefit from better‐equipped hospitals and specialized services, rural areas rely on understaffed and under‐resourced facilities [10, 30, 31, 32]. Socioeconomic inequalities also contribute to unequal access, as low‐income individuals face financial barriers despite the existence of social health protection schemes like the HEF and ID poor, which aim to reduce financial barriers, but coverage and implementation gaps persist [20, 21, 22, 36]. High out‐of‐pocket payments expose low‐income households to catastrophic health expenditures. Vulnerable populations, including disabled individuals, indigenous communities, and migrant workers, remain at heightened risk of receiving substandard care or facing delays in treatment [33, 36]. Addressing these health equity challenges requires stronger governance, targeted resource allocation, and sustained investment in inclusive healthcare models.

## 4 Potential Solutions and Strategies

Strategic investments in healthcare financing, workforce development, infrastructure expansion, and governance reforms are essential to addressing the challenges outlined above. Strengthening regulatory oversight, integrating and improving digital health technologies, and promoting public‐private partnerships are key to enhancing healthcare quality and ensuring equitable access for all Cambodians [8, 9, 10].

### 4.1 Increase Healthcare Financing and Sustainable Investment Strategies

Cambodia's essential health service coverage index stood at 58% in 2021—still below the regional average [3]. Strengthening the health system requires increased financial investment. Improved funding can enhance the availability of medical supplies, the quality of service delivery, and the expansion of primary care services [3, 37]. Scaling up government funding while diversifying revenue sources—such as social health insurance schemes and contributions from NSSF—can support sustainable healthcare financing [1, 7]. According to a 2022 report by the Ministry of Industry, Science, Technology & Innovation, the health sector received 6% of Cambodia's GDP [24]. While this is not an insignificant amount, numerous systemic problems persist. A national initiative for monitoring and evaluating the effective use of healthcare funding is critical to prevent corruption at all levels [1]. Robust governance policies and regulations are required to ensure transparency, rebuild public trust, and move Cambodia's healthcare system closer to international standards.

### 4.2 Strengthen Healthcare Workforce Development and Capacity Building

A shortage of medical personnel remains a barrier to high‐quality healthcare services. Although Cambodia aims to ensure equitable workforce distribution, in 2022 there was an average of 7.4 health personnel per health center—just below the Ministry of Health's recommended standard of 8–10 [3, 6]. Addressing this shortage requires expanding medical education programs, offering competitive salaries, and providing incentives for health workers to serve in rural and underserved areas [8, 37]. Continuing professional development, mentorship programs, and international collaborations can further enhance healthcare workers’ skills and improve retention [38]. Diversifying specializations among healthcare professionals will also strengthen the quality and effectiveness of care.

### 4.3 Strengthen Infrastructure Development

Investing in healthcare infrastructure is necessary to expand access to quality services. Restructuring and upgrading healthcare facilities—especially in remote areas—can help reduce disparities [38]. Equipping hospitals and health centers with modern diagnostic tools, emergency care facilities, and digital technology will improve service efficiency and patient outcomes. Streamlining hospital administration through effective data management systems can reduce wait times and maintain accurate patient records for long‐term care [39, 40]. Expanding and strengthening ambulance services within district health systems can further enhance emergency care access, particularly in remote communities [41].

### 4.4 Enhance Public‐Private Partnerships

As per previous studies carried out by GIZ Cambodia in 2021, the public health care industry in Cambodia is notoriously underpaid, and medical professionals often work for themselves to supplement their income [6]. Relevantly, the World Health Organization reports that the majority of Cambodians seeking medical care now go to private facilities first. The number of private facilities has increased significantly in recent years; in rural areas, the private sector provides approximately 65% of primary care consultations, while in urban areas, it provides between 67% and 78% [6, 20]. While the growth of private clinics has increased service availability, it is also associated with rising out‐of‐pocket payments. Therefore, strategic collaboration between the public and private sectors could enhance service delivery, quality, and innovation. Engaging private providers in government‐led initiatives can expand access, provided that regulatory frameworks are strengthened to ensure quality control, affordability, and alignment with national health goals [8, 30]. Public‐private partnerships can support infrastructure development, workforce training, and the introduction of new medical technologies. Additionally, regulatory frameworks should be strengthened to ensure quality control and affordability in private healthcare services [10, 37].

### 4.5 Leverage Digital Health Technologies

Digital solutions offer promising opportunities to enhance healthcare accessibility and efficiency in Cambodia, but their successful implementation depends on addressing foundational infrastructure gaps. Tools such as telehealth, telemedicine, teleconsultation, and mobile health (mHealth) can extend the reach of essential services, particularly to rural and underserved areas. Implementing electronic health records can also streamline patient data management, reduce administrative burdens, and improve continuity of care [42]. Expanding digital services can bridge access gaps and support disease surveillance, diagnosis, health promotion, and patient engagement [23, 24]. However, many regions still lack internet access, basic digital infrastructure, and limited use of digital literacy, which are prerequisites for digital transformation.

To overcome these barriers, a phased and context‐sensitive approach is essential. In the short term, investments should focus on strengthening digital infrastructure—such as expanding mobile network coverage and internet access—particularly in remote communities. Parallel efforts must include capacity‐building programs to improve digital literacy among healthcare workers and the general population [23, 24]. Long‐term strategies should aim to integrate digital health into the national health system through scalable and sustainable solutions, supported by appropriate policies, funding mechanisms, and inter‐sectoral coordination [10, 24]. While digital health has immense potential, its implementation must be grounded in practical realities. Without addressing the underlying infrastructure and literacy challenges, digital health initiatives risk deepening existing healthcare inequalities rather than reducing them.

### 4.6 Enhance Governance, Policy, and Regulatory Frameworks

Good governance and strong regulatory frameworks are fundamental to health system reform. Cambodia must reform its governance structures to improve healthcare quality and access. Strengthening accreditation and oversight mechanisms for both public and private providers will ensure adherence to service quality standards and patient safety [43]. Decentralizing planning, implementation, and monitoring—along with devolving funding to district health authorities—can enhance responsiveness to local needs, though care must be taken to prevent deepening inequalities [10, 44].

Improved health data management systems are essential for evidence‐based planning, monitoring, and evaluation [44, 45]. Eliminating corruption across all levels of the health system requires strong political will and sustained reform efforts [8]. Establishing a Public Health Commission and enacting a Cambodian Public Health Act could provide a legal framework for regulating healthcare services, filling systemic gaps, and promoting accountability [10]. Transparency in healthcare financing, resource allocation, and expansion of national health insurance schemes will further strengthen system equity and efficiency. Regulatory frameworks should also include stricter licensing, accreditation, and continuous professional development requirements for healthcare professionals [35, 46]. Policies should promote patient‐centered care through feedback mechanisms, safety monitoring, and digital health integration [47]. By enhancing governance and regulatory frameworks, Cambodia can build a more resilient healthcare system that delivers high‐quality, accessible, and affordable care for all citizens.

### 4.7 Strengthen Community‐Based and Preventive Healthcare Initiatives

Strengthening primary healthcare and preventive measures is essential for improving population health and reducing the overall burden on the healthcare system. Community‐based programs such as Village Health Support Groups (VHSGs) and mHealth clinics can improve early disease detection, vaccination coverage, and maternal and child health services—especially in rural and underserved areas [4, 48, 49, 50, 51]. Health education campaigns focusing on disease prevention, hygiene, maternal care, and nutrition empower communities to make informed health decisions [52]. These efforts require adequate funding, proper training for community health workers, and integration with national health programs to ensure sustainability [4, 53, 54, 55, 56]. Preventive efforts should also address risk factors such as poor nutrition, inadequate sanitation, and rising rates of non‐communicable diseases (NCDs) [37, 57]. Public awareness campaigns on hygiene, vaccinations, and healthy lifestyles are crucial and can empower individuals to take charge of their health [52, 58]. Strengthened partnerships between government agencies, NGOs, and communities will expand the reach and impact of these programs [4, 59]. By prioritizing investments in community‐based and preventive healthcare, Cambodia can cultivate a healthier, more resilient population, reduce long‐term healthcare costs, and significantly improve overall health outcomes for all citizens.

## 5 Conclusion

In conclusion, enhancing the quality of health services in Cambodia requires a comprehensive, multi‐pronged approach that addresses challenges in governance, workforce development, financing, and infrastructure. Evidence indicates that strategic interventions—such as public‐private partnerships, capacity‐building initiatives, health equity funds, and the integration of digital health technologies—can significantly improve healthcare access and outcomes. Furthermore, to ensure long‐term sustainability and meaningful progress, systemic reforms are essential; those include strengthening governance, policy, and regulatory frameworks, alongside increasing financial investments. These measures are critical and can significantly contribute to overcoming persistent healthcare challenges and advancing Cambodia's efforts toward achieving UHC.

## Author Contributions

**Virak Sorn:** conceptualization, investigation, writing – original draft, writing – review and editing, validation, formal analysis, data curation.

## Funding

The author has nothing to report.

## Conflicts of Interest

The author declares no conflicts of interest.

## Transparency Statement

The lead author Virak Sorn affirms that this manuscript is an honest, accurate, and transparent account of the study being reported; that no important aspects of the study have been omitted; and that any discrepancies from the study as planned (and, if relevant, registered) have been explained.

## Data Availability

Due to the fact that no data sets were created or examined for this article, data sharing is not applicable.
